# High live birth rates after laparoscopic isthmocele repair in infertility: a systematic review and meta-analysis

**DOI:** 10.3389/fendo.2025.1507482

**Published:** 2025-04-15

**Authors:** Angela Vidal, James Geiger, Vithusha Vinayahalingam, Janna Pape, Marietta Gulz, Tanya Karrer, Michael D. Mueller, Michael von Wolff

**Affiliations:** ^1^ Division of Gynecological Endocrinology and Reproductive Medicine, Women’s University Hospital, Inselspital Bern, University of Bern, Bern, Switzerland; ^2^ Division of Reproductive Endocrinology and Infertility, University Hospital Basel, Basel, Switzerland; ^3^ Department of Gynecology and Obstetrics, Hospital Wolhusen, Luzern, Switzerland; ^4^ Department of Gynecology and Obstetrics, Bern University Hospital, University of Bern, Bern, Switzerland; ^5^ Medical Library, University Library of Bern, University of Bern, Bern, Switzerland

**Keywords:** Cesarean section scar defect, isthmocele, laparoscopic niche resection, reproductive outcomes, Cesarean section

## Abstract

**Background:**

Cesarean sections are becoming more common worldwide. One of the long-term complications of cesarean section is a cesarean scar defect or isthmocele. The presence of isthmocele is associated with infertility.

**Objectives:**

This systematic review and meta-analysis examined the effect of laparoscopic isthmocele repair on the reproductive outcomes of patients with and without infertility.

**Search strategy:**

We searched MEDLINE, EMBASE, and the Cochrane CENTRAL databases in April 2024.

**Selection criteria:**

The study included cohort studies, case-control studies, and case series reporting reproductive outcomes after laparoscopic isthmocele repair among women with or without diagnosed infertility.

**Data collection and analysis:**

The meta-analysis examined rates of live birth, pregnancy, and miscarriage.

**Main results:**

The search identified 866 records and 17 articles were included. Clinical pregnancy rates after isthmocele resection were 62% (95% confidence interval (CI) 54-69%) in women with infertility, compared to 33% (95% CI: 16-57%) in women without infertility and 36% in women with unknown fertility status (36%, 95% CI: 21–55%). Live birth rates were 72% (95% CI: 54–85%) among those with infertility, 78% (95% CI: 46–94%) among those without infertility, and 61% (95% CI: 42–77%) with unknown fertility status. Women with and without infertility had low miscarriage rates of 10% (95% CI: 6–16%) and 7% (95% CI: 3–18%), respectively. The prevalence of co-existing endometriosis was 29% (95% CI: 22–37%). The statistical heterogeneity of the studies ranged from 0 to 86%.

**Conclusions:**

Laparoscopic isthmocele repair has demonstrated the potential to improve reproductive outcomes, specifically in cases where infertility is linked to isthmocele-related factors, such as challenges during embryo transfer or impaired implantation. However, further well-designed multicenter trials must confirm these findings and provide stronger evidence.

**Systematic review registration:**

https://www.crd.york.ac.uk/prospero/, identifier (CRD42024548864).

## Introduction

The prevalence of cesarean deliveries is rising at an alarming rate worldwide (1). The World Health Organization (WHO) has determined that the optimal rate for cesarean delivery is 15% (2, 3), but a global rate of 29% is predicted for 2030. This increase is primarily attributed to the expansion of indications for primary cesarean delivery and a notable decline in vaginal deliveries following a previous cesarean delivery (4).

This increasing cesarean rate is associated with a corresponding increase in short- and long-term complications (5–7). One of the long-term complications is the formation of a cesarean scar defect, which is also referred to as an isthmocele. The European Niche Taskforce has formally defined a cesarean scar defect as a groove in the uterine myometrium of at least 2 mm at the site of the cesarean scar according to transvaginal ultrasound (8, 9). The incidence of Isthmocele is as high as 70% among women who have previously undergone a cesarean section. Approximately 30% of these women experience symptoms (10).

Isthmocele can result in abnormal uterine bleeding, pelvic pain, and ectopic pregnancy (11–13). Furthermore, postmenstrual spotting is associated with Isthmocele volume and is inversely related to residual myometrial thickness (14–16).

One of the most relevant consequences is the risk of infertility. Several studies have shown a decrease of 15-40% in pregnancy and live birth rates following cesarean sections (17–19). In the case of isthmocele, pregnancy, and live birth rates have been reported to be as low as 20%-30%, depending on the size of the defect and whether surgical correction has been attempted (20). Vissers et al. presented potential mechanisms for infertility associated with isthmoceles: Random damage to the environment for sperm penetration and implantation may occur, and intrauterine fluid (mucus or blood) related to the isthmocele may accumulate, potentially hindering implantation. Furthermore, changes in immunobiology or increased inflammation may arise, and distorted uterine contractility may result from fibrosis or disruption of the myometrial layer at the site of the isthmocele, which acts as a physical barrier to embryo transfer and implantation (21).

In recent years, surgical techniques have been developed to treat symptomatic isthmoceles, including laparoscopic excision, resectoscopic, vaginal, and laparotomy repair. Women with isthmocele-associated infertility should be treated individually with a multidisciplinary approach. It has been suggested that isthmocele repair may have a beneficial effect on secondary infertility after cesarean section (22). However, there are no general guidelines for the treatment of isthmocele in cases of infertility.

Laparoscopic surgery offers the additional advantage of diagnosing and treating other potential causes of infertility concurrently (23, 24). In cases where coexisting endometriosis is present, the affected tissue can be resected during the same surgical procedure (25). In some cases, laparoscopy is combined with hysteroscopy, thereby enhancing the visibility of the isthmocele (25, 26).

Currently, there is no conclusive evidence in support of the use of these surgical techniques for reproductive purposes. Therefore, this systematic review and meta-analysis aims to evaluate the results of laparoscopic correction of isthmocele among women with and without infertility and to analyze its impact on reproductive outcomes.

## Materials and methods

### Registration of protocols

The study protocol was registered under the Prospective International Registry of Systematic Reviews, PROSPERO (registry number CRD42024548864). The Preferred Reporting Items for Systematic Reviews and Meta-Analyses (PRISMA) guidelines were used (27).

### Search strategy

A systematic literature search was conducted using the Medline, Embase, and Cochrane CENTRAL databases in April 2024. An initial MEDLINE search strategy was developed by a medical information specialist and tested with a list of basic references. After refinement and querying, complex search strategies were established for each information source based on database-specific controlled vocabulary (thesaurus terms/subject headings) and text words. Synonyms, acronyms, and similar terms were included in the text word search. The search was limited to publications from 1946 to the present. The search terms included “isthmocele”, “niche”, “cesarean section”, “laparoscopic repair”, “Rendez-vous”, and “fertility and pregnancy outcome”. We incorporated respective thesaurus terms and used synonyms, acronyms, and similar terms for all concepts in the text word search. Animal-only studies were excluded from the MEDLINE and Embase searches using a double negative search strategy based on Ovid “humans only” filters. The detailed final search strategies are presented as a **Supplementary File (S1)**. In addition to searching the electronic databases, reference lists and bibliographies of relevant publications were checked for relevant studies. All identified citations were imported into Covidence and duplicates were removed automatically (28).

### Inclusion and exclusion criteria

Investigators AV, JG, and VV independently assessed studies for inclusion using the Covidence software (Covidence systematic review software, Veritas Health Innovation, Melbourne, Australia, www.covidence.org) (29). The eligibility was based on original articles revealing information on reproductive outcomes in patients with or without infertility after laparoscopic resection of isthmocele. Studies that included therapeutic interventions with vaginal techniques or hysteroscopic or repair by laparotomy, as well as studies with an inadequate design or based on animals were excluded.

### Data extraction

The extracted data were abstracted and reviewed in detail by three investigators (AV, JG, and VV) independently. Primary variables of interest included study population characteristics such as patient age, cause and duration of infertility, niche size, pre- and post-intervention RMT, duration of follow-up, presence of endometriosis, and reproductive outcomes (conception method, clinical pregnancy rate, miscarriage rate, and live birth rate). Disagreements were discussed and resolved by consensus.

### Selected groups

The general focus of this study was on women with a desire to become pregnant and a diagnosis of an isthmocele. The population was divided into three groups. The first group comprised women with infertility. Infertility is internationally defined by the World Health Organization (WHO) as the inability to conceive after 12 months of regular, unprotected sexual intercourse. This definition was adopted for its global reach and for representing a well-established clinical and epidemiological standard, taking into account the discrepancies among the clinical guidelines of the American Society for Reproductive Medicine (ASRM), the National Institute for Health and Care Excellence (NICE), and ESHRE (30–32). The second group comprised women who wanted to conceive but did not have infertility, and the third group comprised women with reported results and unknown fertility status.

### Outcomes

Only studies that assess one or more of the following reproductive outcomes were included (32): clinical pregnancy (CP), miscarriage (MC), and live birth (LB). Clinical pregnancy was defined as pregnancy documented by ultrasound with a gestational sac in the uterus. Miscarriage was defined as the spontaneous loss of a fetus before 20 weeks of gestation, and live birth was defined as a delivery that resulted in a live newborn.

### Quality assessment

The Newcastle-Ottawa Scale (NOS) was used for the quality assessment of each study (33). Three parameters were considered for each study: subject selection (0-4 stars), comparability (0-2 stars), and study outcome (0-3 stars). The scoring was as follows: Good quality (= 3 or 4 stars in the selection domain AND 1 or 2 stars in the comparability domain AND 2 or 3 stars in the outcome/exposure domain), fair quality (= 2 stars in the selection domain AND 1 or 2 stars in the comparability domain AND 2 or 3 stars in the outcome/exposure domain), and poor quality (= 0 or 1 star in the selection domain OR 0 stars in the comparability domain OR 0 or 1 stars in the outcome/exposure domain). All included studies were reviewed by AV and VV Independently to assess the risk of bias. Disagreements were resolved by consensus.

### Data synthesis

The primary outcome of our systematic review was the reproductive outcomes (CP, M, LB) after laparoscopic isthmocele repair. For the pooled ORs, statistical analyses were performed with the “metaphor” function of the R software (R Core Team, Vienna, Austria, 2013). Heterogeneity was examined using Cohen’s Q statistic and the I2 statistic. In the presence of high heterogeneity, random-effects models were used.

## Results

### Results of the systematic review

**Figure 1:**
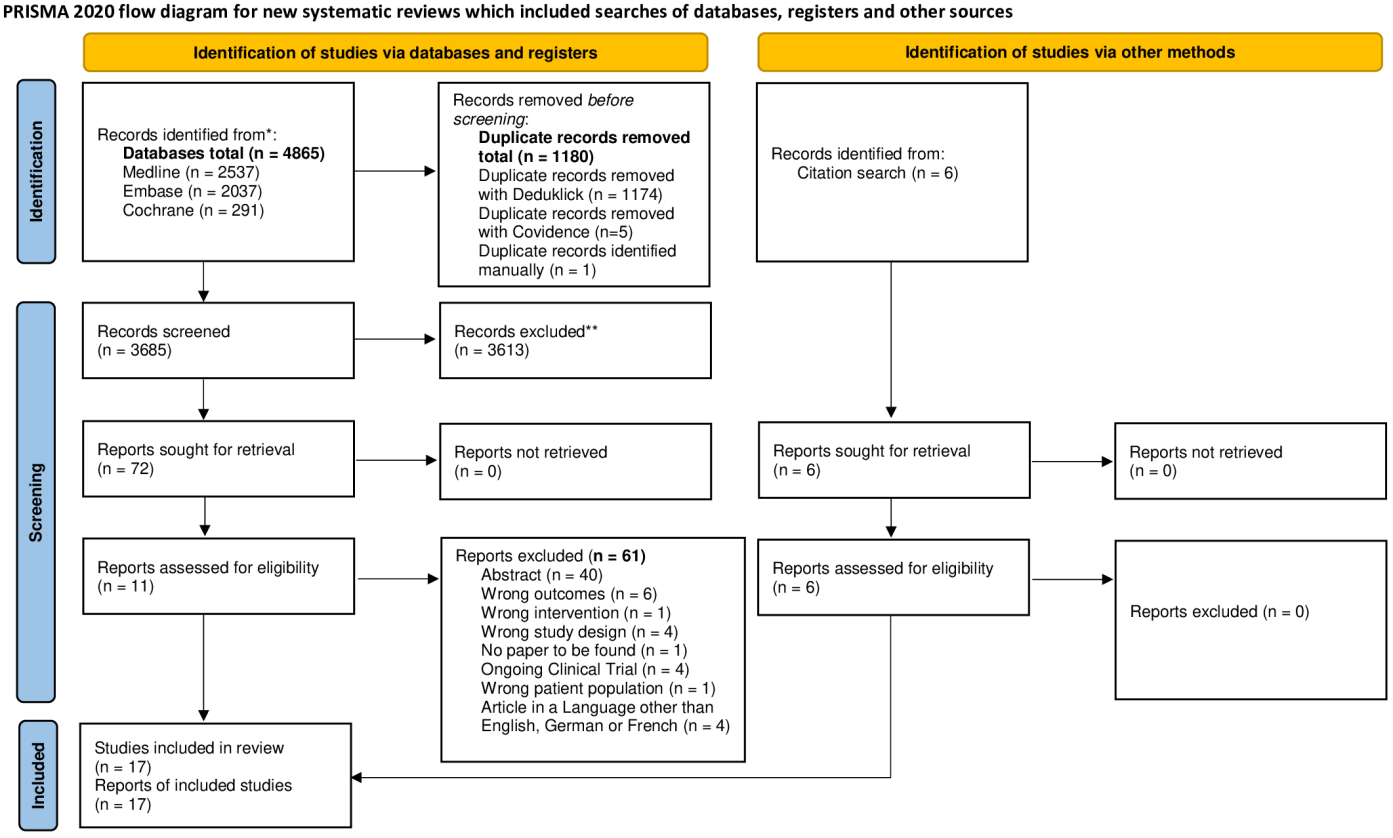
PRISMA flow diagram. FLOWCHART of the literature search and selection process. *Consider, if feasible to do so, reporting the number of records identified from each database or register searched (rather than the total number across all databases/registers). **If automation tools were used, indicate how many records were excluded by a human and how many were excluded by automation tools. Source: Page MJ, et al. BMJ 2021;372:n71. doi: 10.1136/bmj.n71. This work is licensed under CC BY 4.0. To view a copy of this license, visit https://creativecommons.org/licenses/by/4.0/.

A total of 3685 citations were identified from searching the databases. Seventy-eight studies remained after screening the abstracts and full text of the study topic. However, we excluded 61 of these studies for failing to meet our pre-specified inclusion criteria. Therefore, 17 articles were included in the systematic review (**Figure 1**).

### Study characteristics

The characteristics of the study populations are summarized in **Table 1**. The included studies were retrospective (n = 14) and prospective (n = 3). They were conducted in different regions, including Europe (n = 8), America (n = 1), and Asia (n = 8). In total, 866 women were included in the review, of whom 309 (35.6%) (17 studies) were eligible for meta-analysis. The sample sizes of the studies varied considerably, ranging from 9 to 146 patients.

**Table 1:** Characteristics of the included studies (cohorts and case series).

First author, Year of publication	Country	Study design	Total Study population	Number of participants either with LSC+HSC or LSC only	Age, years (mean ± SD)	Previous number of CS	ART before surgical intervention/Total participants and %	Diagnostic done before surgery and after the surgery	Size of the niche (mm) Mean, SD or median (range)	RMT before intervention (mm) Mean, SD or median (range)	RMT post intervention (mm) Mean, SD or median (range)	Postoperative diagnostic	Recommendation to wait before conceiving after surgery (months)	Duration of follow-up (mo) (range)	Mean time since last CS (months)	Endometriosis	Duration of follow-up (months) (range)	Inclusion criteria for LSC/LSC+HSC
Li et al., 2014 (50)	China	Retrospective study	41	17 (LSC)	34,8±4	1(n=31, davon n=10 Emergency section), 2 (n=10)	NM	TVS+HSC	11,4	2,3	9,5	TVS	NM	16	NM	NM	16	RMT<3,5mm + Defect >50% of the anterior uterine wall+desire to get pregnant in the future; RMT<2,5mm + >80% of the anterior uterine wall
Donnez et al., 2017 (51)	France	Observational study with prospective evaluation	38	38 (LSC)	32,6 ±5,6	1(n=25), 2(n=12), 3(n=1)	NM	MRT, TVSS; 3 month post-surgery, Immunhisto und Patho	NM	TVS: 1,7±1 median: 1,6 mm, range 0-3,2 mm, MRI 1,4±0,7mm, median: 1,5 mm range 0-2,7 mm	MRI 9,6±1,8mm, median 9,5 mm, range 5-11,6 mm	MRI	3	12-72	NM	8/38 (21%)	72	RMT < 3mm (MRI). Symptoms: bleeding, pain, infertility
Zhang, X. et al., 2016 (37)	China	Prospective study	142	86 (LSC + HSC)	–	NM-min.1	NM		NM	NM	Not measured	Not measured		28,6(12-45)	24	NM	NM	CSD > 1cm Distance uterine serosa and diverticulum <5mm. RMT<3mm
Delaine et al., 2017 (34)	France	Case Series	9	9 (LSC + HSC)	35 (28-41)	NM – min.1	2/9 (22,2)	TVS/SIS/MRI/HSC/Hormone status	24x16,5x16	1,25 (median – only) available for n=6)	Not measured	Not measured	NM	28	36 (12-84)	3/9 (33,3%)	28	
Zhang, X. et al., 2017 (41)	China	Retrospective study	146	146 (LSC + HSC)	Complete repair: 26,8±2,8 Incomplete repair: 27,1±3,7	NM – min.1	NM	TVS, MRI, HSC, Cur, for irregular bleeding (n=43) in the incomplete group and n=98 in the complete group	Complete repair: 10,3±4,2 Incomplete repair 13,6±6,1	3,8±3,4(incomplete), 3,8±1,7(complete)	Not measured	Not measured	NM	41,1±11,1	NM	NM	41.1	Symptomatic ishtmocele, diagnosis with TVS/MRI/HSC. Only study where RMT > 3 mm was included
Lv et al., 2018 (39)	China	Retrospective study	82	30 (LSC+HSC)	31,2	NM – min. 1	NM	TVS	10,1(6-3)	0,9(0,1-3,9)	Not measured	Not measured	12	21 (13-36)	27	NM	21	Min. 1 CS Sonographic diagnosed isthmocele
Zhang, D. et al., 2019 (38)	China	Retrospective cohort study	67	36(LSC+HSC)	33,5	NM – min.1	NM	TVS	W: 1,4±0,5, L: 0,9±0,3	1,81±0,93	4,68	TVS	12		NM	21/67(31)	NM	
Zhang, N. et al., 2021 (53)	China	Retrospective Cohort Study	62	27(LSC+HSC)	32,6	NM – min.1	NM	TVS und confirmed with SIS	8,2x6,8x6,7	3,1	Not measured	Not measured	NM	18	NM	NM	18	Symptomatic isthmocele TVS diagnosed RMT<3mm Desire future pregnancy
Zhang, Y. et al., 2020 (54)	China	Retrospective Study	65	45(LSC+HSC)	30,2±4,4	1(n=50), 2(n=14), 3 (n=1)	NM	TVS, MRI	W MRI: 19,3±6,4 W TVS: 20,4±7,8	3,26±2,68	Effectiveness 95% no specific data available	TVS	NM	NM	NM	NM	NM	
Karampelas et al., 2021 (35)	Belgium	Retrospective Case series	31	31(LSC)	34,3(24-48)	NM – min.1	NM	TVS/SIS/MRI	NM	1,77±0,86	7,8±1,22	SIS	NM	NM	NM	NM	NM	Symptomatic isthmocele with RMT < 5mm. CS-pregnancy with desire to get pregnant in the future also included
Cardaillac et al., 2023 (40)	France	Retrospective cohort study	33	33(LSC+HSC)	32,6±3,2	1 (n=25), 2(n=7), 3 (n=1)	NM	TVS	NM	1,5	4,26	TVS	NM	24	4,2±1,2 years	NM	29	Isthmocele >20 mm, RMT <3 mm, symptomatic ishtmocele and desire to get pregnant
Gulz et al., 2022 (25)	Switzerland	Retrospective study	83	83(LSC+HSC)	34	NM – min.1	NM	TVS	NM	NA	Not measured	FU(interview and telephone)	3	NM	NM	19/83(22,8)	NM	Symptomatic ishtmocele
Jordans et al., 2022 (9)	Netherlands	Prospective cohort study	100	61(LSC+HSC)	34,5±3,5	1(n=49), 2(n=10), 3 (n=2)	NM	TVS	NM	1(0,6-1,8)	GA 12 - 5,3 (3,8-9,0), GA 20 4,8 (2,7-7,8), 2,2 (1,6 - 4,8)	TVS, niche evaluated 1 Trim: n=54 2 Trim: n= 53 3 Trim: n=51	NM	NM	NM	NM	NM	RMT<3mm, symptomatic niche (primarily fertility problems) but also AUB, pelvic pain, dysmenorrhea
Peng et al., 2022 (52)	China	Retrospective study	24	23(LSC+HSC)	MFFS(35,9±2,3), FSG(32,6±5,2)	Muscle flap filling suture 1,1±0,3, Folding suture group 1,2±0,4)	NM	TVS	NM	MFFS 2,1±1,4, FSG 1,8±0,9	MFFS 6,7±1,8, FSG 6,3±1,7	TVS	NM	3-30	NM	NM	NM	Sonographic diagnosis of ishtmocele Desire to get pregnant RMT < 3mm Symptomatic niche
Fatehnejad et al., 2023 (42)	Iran	Retrospective cohort study	99	45(LSC)	38,4±4,7	1,47±0,55	NM	TVS	8,8(6,9-9,9)	TVS: 2,4(1,75-3,1)	Not measured	FU: interview	NM	12	29,8±4,4	NM	12	Symptomatic ishtmocele RMT < 3mm Failed HSC intervention
Nezhat et al., 2023 (36)	USA	Retrospective study	27	23(LSC+HSC)	36(27-45)	1(n=20), 2 (n=6), 4(n=1)	25/45(55,5)	TVS/MRI/SIS/HSC	NM	NM	Not measured	FU: interview	NM	1-36	NM	12/27(44)	36	Symptomatic niche TVS/SIS/MRI diagnosed niche HSC confirmed niche
Vissers et al., 2023 (18)	Netherlands	Prospective cohort study	133	133(LSC+HSC)	34±3,7	1(1-2)	17/23(73,9)	TVS	9,9(7,5-14,2)	1(0,4-1,7)	Not measured	TVS	6	24	46	NM	24	Niche in the CS scar RMT < 3mm Desire to become pregnant Symptoms: postmenstrual spotting, midcycle intrauterine fluid accumulation, difficulties with a previous embryo transfer

LSK, Laparoscopic; HSK, Hysteroscopic; ART, Artificial Reproductive Technology; CS, cesarean section; NM, not mentioned; IVF, In vitro fertilization; IUI, Intrauterine insemination; RMT, Residual Myometrial Thickness.

We identified one good-quality study (18). The methodological quality of the majority of these studies was rated as either poor (n = 13) or fair (n = 3), mainly due to the lack of a comparison group (**Table 2**).

**Table 2:** Newcastle-Ottawa quality assessment form for cohort studies.

First author, Year of publication	Representativeness of exposed cohort	Selection of non-exposed cohort	Ascertainment of exposure	Outcome of interest not present at study start	Comparability of cohorts on the basis of the design or analysis controlled for confounders	Assessment of outcome	Sufficient length of follow-up for outcomes to occur	Adequacy of follow-up of cohorts	Total	Quality Assessment
Li et al., 2014 (50)	*	–	*	–	–	*	–	*	3/8	poor
Donnez et al., 2017 (51)	*	–	*	*	–	*	*	–	5/8	poor
Zhang, X et al., 2016 (37)	*	–	*	*	–	*	*	–	5/8	poor
Delaine et al., 2017 (34)	*	–	*	–	–	*	*	*	5/8	poor
Zhang, X. et al., 2017 (41)	*	–	*	–	–	*	*	–	4/8	poor
Lv et al., 2018 (39)	*	–	*	*	–	–	*	–	4/8	poor
Zhang, D. et al., 2019 (38)	*	–	*	–	*	–	*	*	4/8	poor
Zhang, N. et al., 2021 (53)	–	–	–	–	*	–	*	*	3/8	poor
Zhang,Y. et al., 2020 (54)	*	–	*	–	–	*	–	–	3/8	poor
Karampelas et al., 2021 (35)	*	–	*	–	–	*	–	–	3/8	poor
Cardaillac et al., 2023 (40)	*	–	*	–	–	*	*	–	4/8	poor
Gulz et al., 2022 (25)	*	–	*	–	*	*	–	*	5/8	fair
Jordans et al., 2022 (9)	–	*	*	–	*	*	–	*	5/8	fair
Peng et al., 2022 (52)	*	–	*	–	–	*	*	–	4/8	poor
Fatehnejad et al., 2023 (42)	*	–	*	–	–	*	–	–	3/8	poor
Nezhat et al., 2023 (36)	*	–	*	–	–	*	*	*	5/8	fair
Visser et al., 2023 (18)	*	–	*	*	*	*	*	*	7/8	good

The included studies vary in design, population size, diagnostic methods, and outcome measures, highlighting the heterogeneity in isthmocele repair literature. The sample sizes range from small case series (*n*=9) (34) to larger prospective cohort studies (*n*=133) (18), reflecting different levels of statistical power. Study populations also differ, with some focusing exclusively on laparoscopic repair (23, 35) and others incorporating combined laparoscopic and hysteroscopy approaches (36, 37).

Of the included articles, 7 studies comprised infertile women with isthmocele (206 women). Five (18, 23, 25, 38, 39) studies described reproductive outcomes in women with a niche without a diagnosis of infertility (118 women) and with unknown fertility status (522 women). Eleven of the studies included a combination of women with and without infertility. Of the 8 studies reporting women with infertility, 2 reported infertility definition or duration. Only 3 studies explicitly examined cases under infertility treatment (**Table 3**). Importantly, only some studies included patients undergoing ART, with notable variations in ART rates (18, 36). Reproductive outcomes among women depending on their fertility status after isthmocele repair surgery are shown in **Tables 3**, **4**.

**Table 3:** Summary results of the included studies: Reproductive outcomes in women with infertility after laparoscopic isthmocele repair.

First author, Year of publication	Time to conceive (mo) Mean (range)	Desire to get pregnant after surgery (Number/ Total) and %	Endometriosis (Number/ Total) and %	Conception mode (Spontaneous) (Number/ Total) and %	Conception mode (IUI) (Number/ Total) and %	Conception mode ( IVF) (Number/ Total) and %	Failed ART (Number/ Total) and %	Pregnant women (Number/ Total number) (%)	Miscarriage (Number/ Total) and %	Live birth (Number/ Total) and %	Delivery mode (Spontaneous (Number/ Total) and %)	Delivery mode (CS) (Number/ Total) and %	Complications during pregnancy/ delivery
Li et al., 2014 (50)	NM	3/3(100)	NM	NM	NM	NM	NM	2/3(66,6)	NM	NM	NM	NM	NM
Donnez et al., 2017 (51)	NM	18/18(100)	6/18(33,3)	NM	NM	NM	NM	8/18(44)	0/8(0)	8/8(100)	0/8(0)	8/8(100)	0
Delaine et al., 2017 (34)	9.5	4/4(100)	NM	3/3(100)	0	0	1/4(25)	3/4 (75)	0	3/3 (100)	0	3/3(100)	0
Karampelas et al., 2021 (35)	NM	12/12(100)	NM	10/10(100)	0	0	NM	10/12(83,3)	NM	6/6	0	6/6	0
Gulz et al., 2022 (25)	NM	40/48(83,3)	11/19(57,8)	NM	NM	NM	NM	25/40(62,5)	2/25(8)	20/25(80)	NM	NM	NM
Nezhat et al., 2023 (36)	NM	15/23(65,2)	NM	9/11(81,8)	0	2/11(18,8)	NM	11/15(73,3)	2/15(13,3)	9/15(60)	1/9(11,1)	8/9(88,88)	0
Vissers et al., 2023 (18)	9	88/88(100)	NM	16/53 (30)	4/53(7,54)	12/53(22,6)	NM	53/88 (60,2)	8/88 (81)	47/53 (88)	4/47(8,5)	43/47(91,4)	2(fetal loss), 3/4 premature delivery

LSK, Laparoscopic; HSK, Hysteroscopic; ART, Artificial Reproductive Technology; CS, cesarean section; NM, not mentioned; IVF, In vitro fertilization; IUI, Intrauterine insemination.

**Table 4:** Summary results of the included studies: Reproductive outcomes in women without infertility and unknown fertility after laparoscopic isthmocele repair.

First author, Year of publication	Time to conceive after Surgery (months)	Desire to get pregnant postoperative (Number/ Total) and %	Pregnant Women (Number/ Total) and %	Conception mode (Spontaneous) (Number/ Total) and %	Conception mode (IUI) (Number/ Total) and %	Conception mode (IVF) (Number/ Total) and %	Miscarriage (Number/ Total) and %	Induced Abortion (Number/ Total) and %	Live Birth (Number/ Total) and %	Pregnant during last Follow-up (Number/ Total) and %	Complications during pregnancy/ Delivery (Number/ Total) and %	Delivery mode (Spontaneous) (Number/ Total) and %	Delivery mode (CS) (Number/ Total) and %
Li et al., 2014 (50)	NM	4/38 (10,4)	4/6(66.6)	NM	NM	NM	NM	NM	5/6(83.3)	1/6(16.6)	NM	NM	NM
Zhang, X. et al., 2016 (37)	NM	32/86 (37,2)	12/32(37,5)	NM	NM	NM	1/12(8,3)	0/12(0)	8/12(66,6)	3/12(25)	NM	NM	NM
Delaine et al., 2016	NM	NM	1/4(25)	1/1(100)	0/1(0)	0/1(0)	0/1(0)	0/1(0)	1/1(100)	0/1(0)	0/1(0)	0/0(0)	1/1(100)
Zhang, X. et al., 2017 (41)	NM	32/146 (21,9)	12/32(37,5)	NM	NM	NM	1/12(8,3)	1/12(8,3)	10/12(83,3)	0/12(0)	NM	0/10(0)	10/10(100)
Lv et al., 2018 (39)	NM	13/30 (43,3)	8/13(61,5)	NM	NM	NM	5/8(62,5)	0/8 (0)	3/8(37,5)	0/8(0)	NM	1/3(33,3)	2/3(66,6)
Zhang, D. et al., 2019 (38)	NM	20/36 (55,5)	10/20(50)	NM	NM	NM	5/10(50)	0/10(0)	5/10(50)	0/10(0)	0/10(0)	1/5(20)	4/5(80)
Zhang, N. et al., 2020	NM	27/27 (100)	8/27(29,6)	NM	NM	NM	2/8(25)	0/8 (0)	6/8(75)	0/8(0)	2/6(33,3)	0/6(0)	6/6(100)
Zhang, Y. et al., 2020 (54)	NM	36/43 (83.7)	15/36(41.6)	NM	NM	NM	1/15(6,6)	0/15(0)	6/15(40)	8/15(53,3)	NM	NM	6/6(100)
Karampelas et al., 2021 (35)	NM	NM	4/19(21,1)	4/4 (100)	0/4(0)	0/4(0)	NM	NM	NM	NM	NM	NM	NM/4
Cardaillac et al., 2023 (40)	10.2	20/27 (74)	15/20(75)	9/15(60)	NM	NM	1/15(6,6)	NM	14/15(93,3)	0/15(0)	NM	NM	NM
Gulz et al., 2022 (25)	NM	NM	NM	NM	NM	NM	NM	NM	NM	NM	7/38(18,4)	NM	NM
Jordans et al., 2022 (9)	NM	NM	61/61(100)	NM	NM	NM	NM	NM	48/61(67.2)	10/61 (16.3)	NM	NM	48/48(100)
Peng et al., 2022 (52)	NM	11/23 (47,8)	10/11(90,9)	NM	NM	NM	3/10(30)	2/10(20)	5/10(50)	0	NM	0/5(0)	5/5(100)
Fatehnejad et al., 2023 (42)	NM	NM	27/45(8,9)	NM	NM	NM	NM	NM	NM	NM	NM	NM	NM
Vissers et al., 2023 (18)	9	45/45 (100)	34/45(75,5)	40/45(88,8)	0/45(0)	5/45(11,1)	4/34 (11,7)	NM	30/34(88,2)	2 lost to follow up	NM	0/45(0)	30/30(100)

LSK, Laparoscopic; HSK, Hysteroscopic; ART, Artificial Reproductive Technology; CS, cesarean section; NM, not mentioned; IVF, In vitro fertilization; IUI, Intrauterine insemination.

Preoperative diagnostic methods varied, with most studies utilizing transvaginal sonography (TVS), while some included additional imaging techniques such as MRI and saline infusion sonohysterography (23, 34, 36). Differences in niche size assessment and residual myometrial thickness (RMT) measurement further complicate comparisons across studies. Some studies reported significant improvements in RMT post-surgery (38, 40), whereas others did not assess this outcome.

Residual myometrial thickness ranged from 2.5 mm to 5 mm in all women who underwent laparoscopic niche repair. The majority of studies have indicated that laparoscopic repair is the optimal approach when myometrial thickness is less than 3 mm, as this reduces the risk of perforating the bladder with the hysteroscopic approach. One study (41) has already established the indication for laparoscopic repair of the niche starting from a myometrium thickness of 5 mm. In three studies (25, 40, 42) the laparoscopic niche repair was conducted due to a cesarean scar pregnancy involving a total of 25 women. The reproductive outcomes of these cases were not described separately.

Endometriosis was reported in several studies, with prevalence rates ranging from 21% to 44% ( (18, 24, 25, 34, 36), suggesting a potential association between isthmocele and endometriosis. However, few studies accounted for its impact on fertility outcomes, leaving gaps in understanding its role in reproductive prognosis. Two studies described findings of iatrogenic adenomyosis in the uterine scar tissue. Gulz et al. reported an overall prevalence of endometriosis of 26.5% (25) with a predominance of peritoneal endometriosis (63%). It is noteworthy that 11% of patients (n=9) exhibited iatrogenic adenomyosis in the uterine scar tissue. The presence of iatrogenic adenomyosis was associated with the co-existence of extrauterine endometriosis (25). Donnez et al. described adenomyosis in the uterine scar with a prevalence of 21% (23).

### Results of the meta-analysis

A meta-analysis of 17 studies comprising 309 women was conducted to evaluate reproductive outcomes in women after laparoscopic niche resection (**Figure 1**). CPR was calculated considering all women who underwent isthmocele repair; LBR was estimated using the population of women who achieved pregnancy as the reference denominator.

### Reproductive outcomes after laparoscopic isthmocele repair

**Figure 2:**
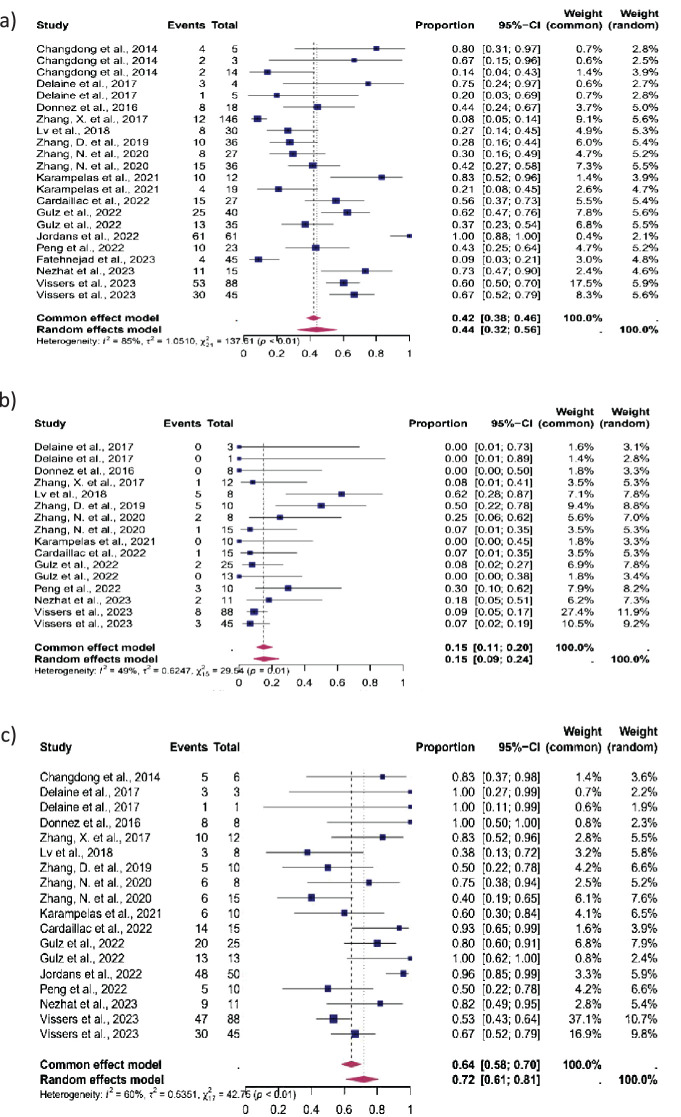
Pooled overall prevalence of the effect of laparoscopic isthmocele repair reproductive outcomes. Forest plot of proportions and 95% confidence intervals (CI) for studies evaluating the prevalence of reproductive outcomes in women who underwent laparoscopic repair of the isthmocele. Blue squares for each study indicate the proportion, the size of the boxes indicates the weight of the study, and the horizontal lines indicate the 95% CI. The data in bold and pink diamond represent the pooled prevalence for post-treatment infertility and 95% CI. Overall estimates are shown in the fixed- and random-effect models. This pooled overall prevalence analysis evaluates three key variables: **(a)** clinical pregnancy rate, **(b)** miscarriage rate, and **(c)** live birth rate.

15 studies were eligible for inclusion in the analysis of CP, 12 in the analysis of MC, and 14 in the analysis of LB. After isthmocele repair, CP occurred in 44% (95% CI: 32-56%), MC in 15% (95% CI: 9-24%) and LB in 72% (95% CI, 61%–81%) (**Figure 2**) The heterogeneity test revealed significant heterogeneity among the studies I^2^ = 85, p < 0.01, I^2^ = 49, p < 0.01 and I^2^ = 60, p < 0.01 respectively.

### Subgroup analysis

**Figure 3:**
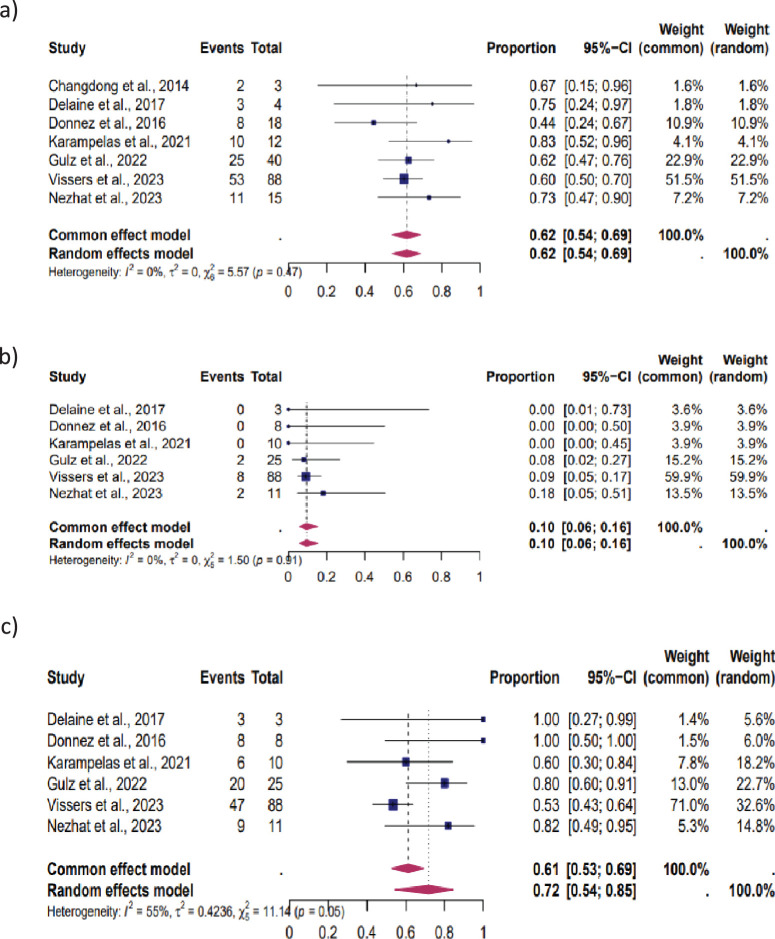
Pooled overall prevalence of the effect of laparoscopic isthmocele repair on reproductive outcomes in women with infertility. For details, see the legend of **Figure 2**. This pooled overall prevalence analysis evaluates three key variables: **(a)** clinical pregnancy rate, **(b)** miscarriage rate, and **(c)** live birth rate.

**Figure 4:**
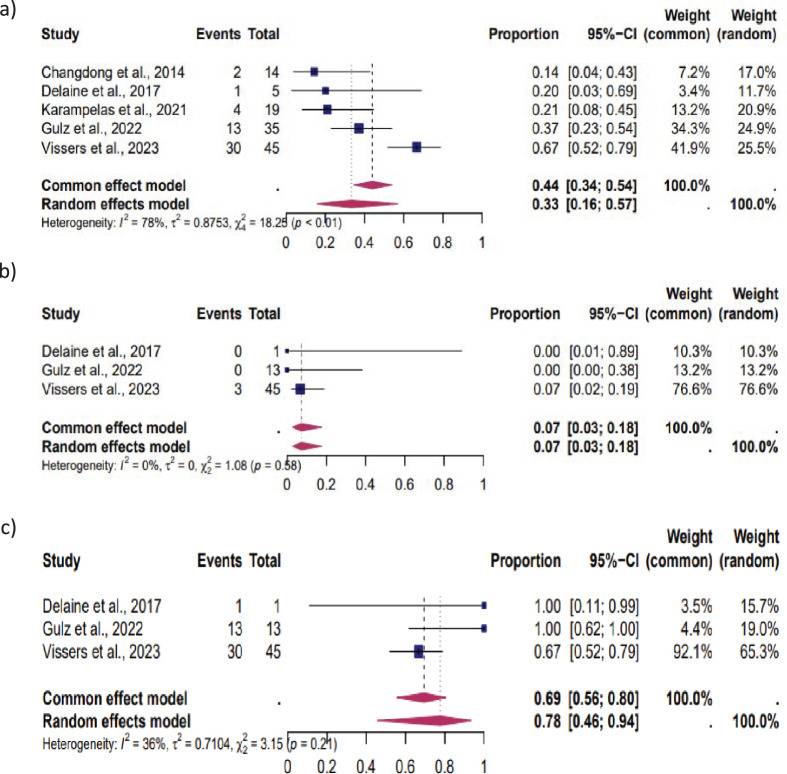
Pooled overall prevalence of the effect of laparoscopic isthmocele repair on reproductive outcome in women without infertility. For details, see the legend of **Figure 2**. This pooled overall prevalence analysis evaluates three key variables: **(a)** clinical pregnancy rate, **(b)** miscarriage rate, and **(c)** live birth rate.

**Figure 5:**
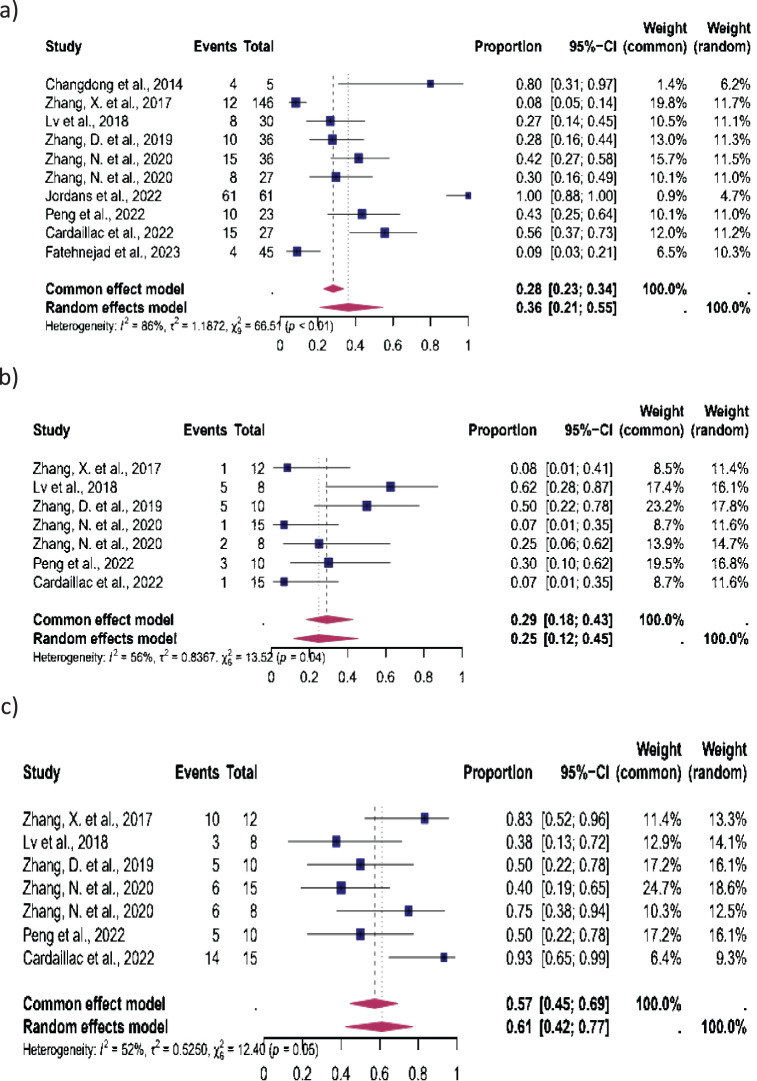
Pooled overall prevalence of the effect of laparoscopic isthmocele repair of the reproductive outcome in women with unknown fertility status. For details, see the legend of **Figure 2**. This pooled overall prevalence analysis evaluates three key variables: **(a)** clinical pregnancy rate, **(b)** miscarriage rate, and **(c)** live birth rate.

The reproductive outcomes were stratified according to the fertility status (women with infertility, without infertility, and unknown fertility status) (**Figures 3**–**5**).

*Women with infertility:* Seven studies were eligible for inclusion in this subgroup analysis: CP was observed in 62% (95% CI, 54%–69%), MC in 10% (95% CI, 6%–16%) and LB in 72% (95% CI, 54%–85%). The heterogeneity test revealed significant heterogeneity among the studies I^2^ = 0, p < 0.01, I^2^ = 0, p < 0.01 and I^2^ = 55, p < 0.01 respectively (**Figure 3**).

*Women without infertility:* Five studies were included in this subgroup analysis, which focused on women without infertility. The results showed that 33% (95% CI, 16%–57%) of these women experienced CP, 7% (95% CI, 3%–18%) MC, and 78% (95% CI, 46%–94%) LB. The heterogeneity test revealed significant heterogeneity among the studies for CP (I^2^ = 78, p < 0.01), MC (I^2^ = 0, p < 0.01), and LB (I^2^ = 36, p < 0.01) (**Figure 4**).

*Women with unknown fertility status:* Ten studies were included in this subgroup analysis. CP occurred in 36% (95% CI, 21%–55%), MC in 25% (95% CI, 12%–45%), and LB in 61% (95% CI, 42%–77%). The heterogeneity test revealed significant heterogeneity among the studies, with I^2^ = 86, p < 0.01 for CP, I^2^ = 56, p < 0.01 for MC, and I^2^ = 52, p < 0.01 for LB (**Figure 5**).

### Prevalence of endometriosis during isthmocele repair

**Figure 6:**
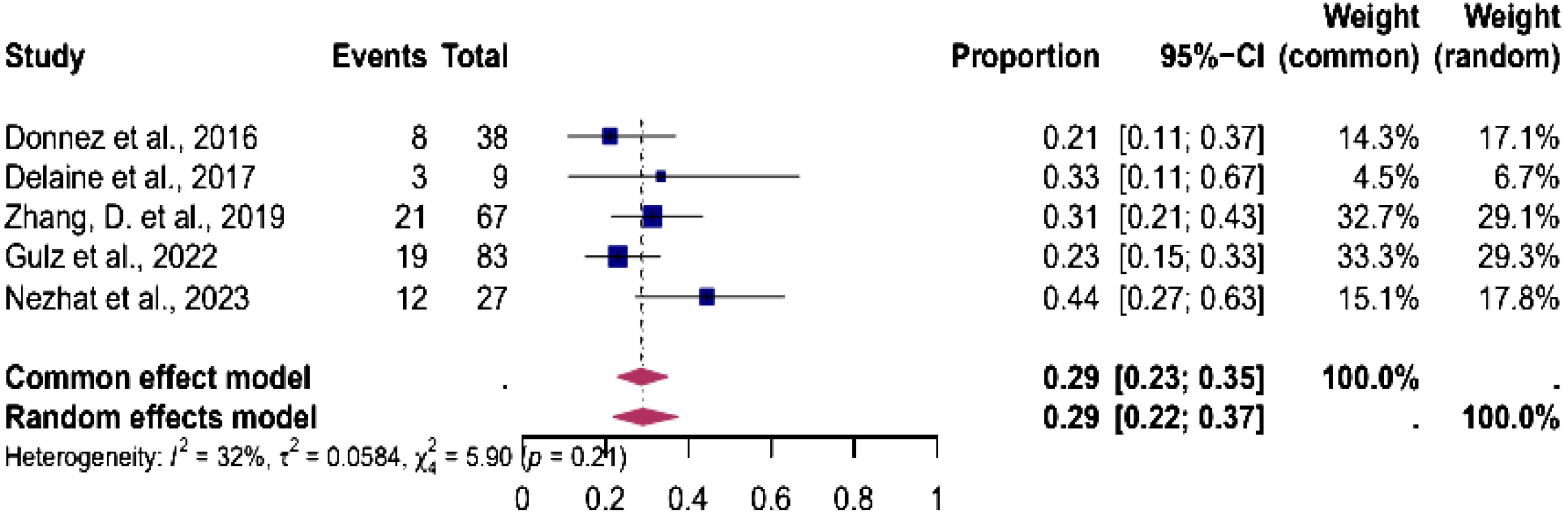
Pooled overall prevalence of endometriosis in women during laparoscopic isthmocele repair. Forest plot of proportions and 95% confidence intervals (CI) for studies evaluating the prevalence of endometriosis in women undergoing laparoscopic isthmocele repair. Blue squares for each study indicate the proportion, the size of the boxes indicates the weight of the study, and the horizontal lines indicate the 95% CI. The data in bold and pink diamond represent the pooled prevalence for post-treatment infertility and 95% CI. Overall estimates are shown in the fixed- and random-effect models. This pooled overall prevalence analysis evaluates three key variables: **(a)** clinical pregnancy rate, **(b)** miscarriage rate, and **(c)** live birth rate.

Five studies were eligible for the analysis of the pooled prevalence of endometriosis diagnosed during isthmocele repair. The analysis showed an overall prevalence of endometriosis of 29% (95% CI, 22%-37%). The heterogeneity test showed low heterogeneity between studies I^2^ = 32, p < 0.01 (**Figure 6**).

## Discussion

### Main findings

The aim of this study was to evaluate the effect of laparoscopic isthmocele repair on reproductive outcomes. Given the recent increase in cesarean section rates and potential long-term complications, considerable attention has been given to evaluating whether surgery has the potential to improve reproductive outcomes among women with isthmoceles.

Our review revealed the positive effect of laparoscopic isthmocele resection on reproductive outcomes, with the following key findings: First, 44% of women experienced CP after isthmocele repair, while LB was notably high at 72%, with all pregnant women as the reference denominator. Second, women with infertility had the highest rates of CP (62%; 95% confidence interval (CI): 54–69%) and LB (72%; 95% CI: 54–85%) compared to women without infertility (CP: 33%, 95% CI: 16–57%/LB: 78%, 95% CI: 46–94%) and women with unknown fertility status (CP: 36%, 95% CI: 21–55%/LB: 61%, 95% CI: 42–77%). Third, the prevalence of endometriosis at surgery was 29% (95% CI: 22–37%).

### Strengths and limitations

Although our study strictly followed the recommendations to provide high-quality evidence summaries, some limitations are evident. First, most of the included studies were based on retrospective data, resulting in high statistical heterogeneity. Second, there was a lack of data regarding inconsistency, poor description of other relevant causes of infertility, no information on the time until pregnancy, and a lack of data regarding fertilization methods. Third, another limitation of the included studies is that they did not exclusively include women with secondary infertility, but also women who presented with difficulties in embryo transfer or had other bleeding disturbances. This broader patient population may introduce variability in the results and limit the ability to generalize the findings specifically to women with secondary infertility. Thus, we could not perform sub-analyses of cases requiring ART. Fourth, some studies did not justify the choice of treatment, and there was a paucity of information regarding the cesarean scar defect or magnetic resonance imaging following surgery.

### Interpretation

Infertility with isthmocele was significantly higher than without (66% vs 46%; p=0.03) (43). Complete niche resection reduces the incidence of infertility-related complications such as postmenstrual bleeding and chronic endometritis. This may be explained by the prevention of blood accumulation, which is associated with disturbed cervical mucus quality, sperm transport, and the uterine microbiota, potentially interfering with the delicate process of embryo implantation (21, 23).

One mechanism that could explain the association between isthmocele and infertility, particularly in patients undergoing ART, is the alteration of the endometrial environment due to hemorrhagic disturbances. Residual and abnormal bleeding caused by the isthmocele creates an environment that is less receptive to embryo implantation, as it interferes with the synchronization between the endometrial phase and embryo transfer. Furthermore, the presence of isthmocele may complicate embryo transfer, further exacerbating the challenges for achieving successful implantation. This disruption affects the quality and stability of the endometrium, significantly compromising reproductive outcomes (21). Therefore, our results suggest that laparoscopic repair of the isthmocele may improve reproductive outcomes by reducing the factors previously described.

Our study revealed that isthmocele repair led to a 44% CPR and a 72% LBR (having all pregnant women as the denominator), suggesting that while implantation may be initially impaired, pregnancy maintenance improves significantly. Endometrial alterations, including residual bleeding and structural anomalies, likely create a less receptive environment for embryo implantation (21). These findings highlight the potential benefits of isthmocele repair in improving reproductive outcomes, particularly by addressing factors that interfere with early implantation and gestational progression (44, 45).

In a recent systematic review and meta-analysis (22), no clear differences were found in the prevalence of CP, MC, and LB between treatment options (laparoscopic suturing and knotting, hysteroscopy, laparotomy, and vaginal approach) (22). In our meta-analysis, however, we found that the prevalence of LB was higher in the infertility group than among women without infertility. One interpretation of these results could be that among women with infertility, the isthmocele may contribute to fertility problems, so the treatment of the niche may have more impact. In addition, the procedure results in enhanced passage and anatomical suitability for embryo transfer, and it also permits the potential for performing endometriosis resection, which is also related to infertility (25).

However, as laparoscopic surgical repair is a non-standardized treatment, the quality of studies on this topic is heterogeneous. Only one included study had good quality (18). The study cohorts had mixed populations and a lack of information on fertility history or the need for any fertility treatment, especially the use of assisted reproductive techniques. Therefore, a sub-analysis of the group of patients receiving assisted reproductive treatment could not be performed.

There is a complete lack of randomized controlled trials comparing the effect of isthmocele repair on reproductive outcome parameters with control groups without surgical intervention. So far, the LAPRES trial (Dutch Trial Register (ref. no. NL6350 http://www.trialregister.nl). Is the only registered trial in which patients with infertility have undergone laparoscopic repair. This is a randomized, unblinded, controlled trial involving 200 infertile women with a 2-year follow-up (46).

Reproductive outcome analysis in infertile patients after isthmocele repair, as performed in our study, is currently the only strategy to assess the impact of surgical interventions on reproductive outcomes. Notably, none of the selected studies specifically reported on the mode of conception of subsequent pregnancy among women with previous failed ART before surgery. Nezhat et al. (36) and Vissers et al. (18) included patients undergoing ART, a key factor in evaluating the impact of isthmocele repair on reproductive outcomes. However, neither study specified the mode of conception in women with prior ART failures before surgery, making it difficult to determine whether improved outcomes were due to the procedure itself or ART. Further research with robust methodological design is needed to control for these factors and clarify the association between surgical intervention and reproductive outcomes.

Five studies advised patients to wait at least three months before attempting conception after surgery. Conversely, 2 studies suggest a waiting period of 1 year before trying again (25, 38). Only the study by Vissers et al. (18), included recommendations for postoperative care, which entailed the administration of contraceptives for 6 months following the procedure. This was deemed necessary to allow for sufficient postoperative time for uterine healing. Notably, none of the studies identified any perioperative complications, especially a lack of uterine dehiscence described after other isthmocele interventions. None of the studies mentioned postoperative complications.

A key aspect of the surgical approach is that laparoscopy has the added advantage of simultaneous diagnosis and treatment of other potential causes of infertility (24). Endometriosis is often a co-morbidity and can cause infertility, chronic inflammation, and anatomical changes due to adhesions. The role of endometriosis as a risk factor for isthmocele is still not fully established, but emerging evidence suggests a potential association between the two conditions. Endometriosis could impair post-cesarean wound healing, leading to defective scar formation and increasing the risk of isthmocele. This may occur through chronic inflammation, fibrosis, altered immune responses, and abnormal endometrial remodeling, all of which are known to affect tissue repair. Proposed mechanisms include altered endometrial receptivity, intrauterine fluid accumulation, disrupted uterine contractility, and a higher prevalence of recurrent implantation failure (21, 36, 47).

Endometriosis was found in 27% of patients with isthmocele who underwent laparoscopic resection in a retrospective study by Gulz et al. (25). The findings of the Gulz et al., 2022 study, which reported a 27% prevalence of endometriosis, align closely with our results, indicating a 29% prevalence and further supporting the association between isthmocele and endometriosis (25). The presence of endometrial glands or stromal tissue within the scar was found in 21-27% of cases in two other studies (23, 25, 48). A deeper understanding of this association is essential to optimize treatment strategies and improve patient outcomes. Therefore, endometriosis can be resected during the same procedure. This suggests a potential association between isthmocele and endometriosis, warranting further research to clarify the underlying mechanisms and clinical implications of this relationship.

Considering the availability of different treatment options and the lack of clinical guidelines on this issue, consideration should be given to ultrasound of the residual myometrial thickness, the presence of other pathologies (e.g., endometriosis, adhesions, tubal obstruction, etc.), as well as the patient’s symptoms before deciding on a management approach. If surgical treatment is indicated, the choice between a hysteroscopic resection and laparoscopic or vaginal repair should be based on factors such as the residual myometrial thickness and the skills of the surgeon (49). If the residual myometrial thickness is less than 3 mm, a laparoscopic or vaginal repair technique should be used. The defect is completely removed, and the myometrium is reattached with sutures.

Furthermore, Verberkt et al. recommended that future studies should examine the effects of uterine-niche-related surgery (22): Important topics include structured evaluation of all causes of infertility, cesarean scar defect measurement before and after surgery, structured follow-up for at least 2 years, detailed information on duration of interest, previous fertility treatments or about conception mode, and sample size powered for pregnancy rate. Considering these recommendations and interdisciplinary work between surgeons, obstetricians, and reproductive medicine, the underlying role of infertility and its outcomes in terms of reproduction can be identified.

## Conclusion

Laparoscopic repair of isthmoceles is associated with good reproductive outcomes, suggesting this intervention is effective. Women with a history of infertility may benefit. However, further Randomized Controlled Trials are required to provide robust evidence to support this hypothesis.

## Data Availability

The datasets presented in this study can be found in online repositories. The names of the repository/repositories and accession number(s) can be found in the article/**Supplementary Material**.

## Supplementary material

Supplementary Table 1 Database Search Strategies. A systematic literature search in Medline, Embase, and Cochrane CENTRAL.
