# Supplementary material for: High live birth rates after laparoscopic isthmocele repair in infertility: a systematic review and meta-analysis
Source: Front Endocrinol (Lausanne). 2025 Apr 15;16:1507482. doi: 10.3389/fendo.2025.1507482 (PMC12037975; doi:10.3389/fendo.2025.1507482)
Supplement: Supplementary Table 1 — Database Search Strategies. A systematic literature search in Medline, Embase, and Cochrane CENTRAL. [file DataSheet1.docx]

**Impact of uterine niche resection on fertility, pregnancy outcomes, and artificial reproduction techniques**

Principal Investigators: Dr. med. Angela Vidal
Information Specialist: Tanya Karrer

**Detailed search strategy**

To identify potentially relevant publications on the topic, a search strategy was designed, and investigated in Embase, MEDLINE, and Cochrane Library. The core concepts of the search strategy consisted of 1. Isthmocele/cesarean scar 2. Resection, 3. Artificial reproduction techniques, and 4. Fertility and pregnancy outcome. A medical information specialist developed an initial search strategy in Embase and tested it against a list of core references to ensure key publications were included. After refinement, the information specialist set up the search strategy for each information source based on database-specific index terms and free text. The free text search included synonyms, acronyms, and similar terms. In the Cochrane Library results were filtered for reviews and trials. No other database-provided limits have been applied in any sources considering any other formal criteria. Studies concerning exclusively animals were excluded from the searches by using a double-negative search strategy based on the "Humans only" filters by Ovid. The search was finalized on 24/04/2024. The results were deduplicated using the automated deduplication tool of Covidence.
Screening, data extraction, and study assessment took place in Covidence.

| **Search date** | **Database searched** | **Platform** | **Dates of coverage** | **Records** |
| --- | --- | --- | --- | --- |
| 24 April 2024 | Embase | Ovid | 1974 - 2024 April 22 | 2037 |
| 24 April 2024 | Medline ALL | Ovid | 1946 - 2024 April 23 | 2537 |
| 24 April 2024 | Cochrane Database of Systematic Reviews | Wiley | 1992 - present | 9 |
| 24 April 2024 | Cochrane Central Register of Controlled Trials | Wiley | 1992 - present | 282 |
|  |  |  | Sum of references | 4’865 |
|  |  |  | Removed duplicates | -1180 |
|  |  |  | **Total references** | **3685** |

**Search strategy used per database**

**Embase <1974 to 2024 April 22>**

Search date: 24 April 2024

1 (((C?esar* or fetectomy or postc?esar* or post-c?esar* or (abdominal adj3 deliver*)) and (niche*1 or scar*1 or scarring or dehiscen* or diverticul*2 or pouch*2 or uterine defect*1 or keloid*1 or cicatrix or cicatrices)) or uterine niche*1 or isthmocel* or istmocel* or uterine defect*1).ti,ab,kw. 6807

2 uterus disease/ and cesarean section/ 934

3 1 or 2 7479

4 (laparoscop* or hysteroscop* or rendez-vous or rendezvous or isthmoplast* or endoscop* repair* or (robot* adj3 surger*) or folding sutur* or muscle flap filling sutur* or transvaginal or isthmocele surger*).ti,ab,kw. 305873

5 (exp hysteroscopy/ or exp laparoscopy/ or exp robot assisted surgery/ or exp laparoscopic surgery/ or obstetric operation/) and (exp keloid/ or exp hypertrophic scar/ or exp atrophic scar/ or exp scar formation/ or scar/ or exp wound dehiscence/) 4384

6 4 or 5 306731

7 (assisted reproduction* or (fertil* adj3 therap*) or (fertil* adj3 treat*) or (infertil* adj3 therap*) or (infert* adj3 treat*) or IUI or Articificial inseminat* or Intrauter* inseminat* or Uterine inseminat* or Intracytoplasmic Sperm Inject* or ICSI or ivf or in-vitro fertili#ation* or invitro fertili#ation* or embry* transfer* or embry* implant* or blastocyt* implant* or inseminat* or ((semen or sperm) adj2 (deposit* or inject*))).ti,ab,kw. 143564

8 infertility therapy/ or artificial insemination/ or embryo disposition/ or gamete intrafallopian transfer/ or in vitro fertilization/ or oocyte donation/ 67664

9 (obstetrical outcome*1 or pregnan* or fertil* or deliver* or conception or conceive or reproductive outcome*1 or gestation* or gravid* or Labo?r or Parity or parities or Birth* or Parturit* or Childbirth* or perinatal mortality or nidation* or fecund* or subfecund* or sub-fecund* or infecund* or infertil* or subfert* or sub-fert* or anovularit* or gonad* or reproduction* or Ovari* or ovary or ovulat* or folliculo* follicular or oogenes#s or oocyte* or (parous or primipar* or multipar* or primigravid* or multigravid* or perinatal* or peripartum or peri partum or prepartum or pre partum or intrapartum or intra partum or postpartum or post partum or childbear* or ((vaginal or abdominal) adj2 delivery) or caesarean* or cesarean* or c-section* or embryo* or f?etus* or f?etal* or placenta* or prematur* or stillbirth* or abort* or congenital*)).ti,ab,kw. 3857559

10 exp reproduction/ or exp "parameters concerning the fetus, newborn and pregnancy"/ 1643548

11 or/7-10 4357249

12 and/3,6,11 2039

13 (exp animal/ or exp invertebrate/ or nonhuman/ or animal experiment/ or animal tissue/ or animal model/ or exp plant/ or exp fungus/) not (exp human/ or human tissue/) 7904178

14 12 not 13 2037

******************

**Ovid MEDLINE(R) ALL <1946 to April 23, 2024>**

Search date: 24 April 2024

1 (((C?esar* or fetectomy or postc?esar* or post-c?esar* or (abdominal adj3 deliver*)) and (niche*1 or scar*1 or scarring or dehiscen* or diverticul*2 or pouch*2 or uterine defect*1 or keloid*1 or cicatrix or cicatrices)) or uterine niche*1 or isthmocel* or istmocel* or uterine defect*1).ti,ab,kw. 4321

2 Uterine Diseases/ and Cesarean Section/ 651

3 1 or 2 4851

4 (laparoscop* or hysteroscop* or rendez-vous or rendezvous or isthmoplast* or endoscop* repair* or (robot* adj3 surger*) or folding sutur* or muscle flap filling sutur* or transvaginal or isthmocele surger*).ti,ab,kw. 189076

5 Hysteroscopy/ or exp Laparoscopy/ or Robotic Surgical Procedures/ or Obstetric Surgical Procedures/ or cicatrix/ or cicatrix, hypertrophic/ or keloid/ or Surgical Wound Dehiscence/ 178343

6 4 or 5 255612

7 (assisted reproduction* or (fertil* adj3 therap*) or (fertil* adj3 treat*) or (infertil* adj3 therap*) or (infert* adj3 treat*) or IUI or Articificial inseminat* or Intrauter* inseminat* or Uterine inseminat* or Intracytoplasmic Sperm Inject* or ICSI or ivf or in-vitro fertili#ation* or invitro fertili#ation* or embry* transfer* or embry* implant* or blastocyt* implant* or inseminat* or ((semen or sperm) adj2 (deposit* or inject*))).ti,ab,kw. 96665

8 exp Reproductive Techniques, Assisted/ 81908

9 (obstetrical outcome*1 or pregnan* or fertil* or deliver* or conception or conceive or reproductive outcome*1 or gestation* or gravid* or Labo?r or Parity or parities or Birth* or Parturit* or Childbirth* or perinatal mortality or nidation* or fecund* or subfecund* or sub-fecund* or infecund* or infertil* or subfert* or sub-fert* or anovularit* or gonad* or reproduction* or Ovari* or ovary or ovulat* or folliculo* follicular or oogenes#s or oocyte* or (parous or primipar* or multipar* or primigravid* or multigravid* or perinatal* or peripartum or peri partum or prepartum or pre partum or intrapartum or intra partum or postpartum or post partum or childbear* or ((vaginal or abdominal) adj2 delivery) or caesarean* or cesarean* or c-section* or embyro* or f?etus* or f?etal* or placenta* or prematur* or stillbirth* or abort* or congenital*)).ti,ab,kw. 3111754

10 exp Reproduction/ or Pregnancy Outcome/ or pregnancy rate/ 1277260

11 or/7-10 3487851

12 and/3,6,11 2549

13 (exp animal/ or exp invertebrate/ or animal experiment/ or animal model/ or exp plant/ or exp fungus/) not exp human/ 5653346

14 12 not 13 2537

******************

**Cochrane Database of Systematic Reviews and Central Register of Controlled Trials**

Search date: 24 April 2024

#1 (((C?esar* or fetectomy or postc?esar* or post-c?esar* or (abdominal NEAR/3 deliver*)) and (niche* or scar* or scarring or dehiscen* or diverticul* or pouch* or (uterine NEXT defect*) or keloid* or cicatrix or cicatrices)) or (uterine NEXT niche*) or isthmocel* or istmocel* or (uterine NEXT defect*)):ti,ab,kw 868

#2 [mh ^"Uterine Diseases"] AND [mh ^"Cesarean Section"] 7

#3 #1 OR #2 872

#4 (laparoscop* or hysteroscop* or rendez-vous or rendezvous or isthmoplast* or endoscop* repair* or (robot* NEAR/3 surger*) or folding sutur* or (muscle NEXT flap NEXT filling NEXT sutur*) or transvaginal or (isthmocele NEXT surger*)):ti,ab,kw 34069

#5 [mh ^Hysteroscopy] OR [mh Laparoscopy] OR [mh ^"Robotic Surgical Procedures"] OR [mh ^"Obstetric Surgical Procedures"] OR [mh ^cicatrix] OR [mh ^"cicatrix, hypertrophic"] OR [mh ^keloid] OR [mh ^"Surgical Wound Dehiscence"] 12559

#6 #4 OR #5 36762

#7 ((assisted NEXT reproduction*) or (fertil* NEAR/3 therap*) or (fertil* NEAR/3 treat*) or (infertil* NEAR/3 therap*) or (infert* NEAR/3 treat*) or IUI or (Articificial NEXT inseminat*) or (Intrauter* NEXT inseminat*) or (Uterine NEXT inseminat*) or (Intracytoplasmic NEXT Sperm NEXT Inject*) or ICSI or ivf or (in-vitro NEXT fertili?ation*) or (invitro NEXT fertili?ation*) or (embry* NEXT transfer*) or (embry* NEXT implant*) or (blastocyt* NEXT implant*) or inseminat* or ((semen or sperm) NEAR/2 (deposit* or inject*))):ti,ab,kw 15222

#8 [mh "Reproductive Techniques, Assisted"] 4605

#9 ((obstetrical NEXT outcome*) or pregnan* or fertil* or deliver* or conception or conceive or (reproductive NEXT outcome*) or gestation* or gravid* or Labo?r or Parity or parities or Birth* or Parturit* or Childbirth* or (perinatal NEXT mortalit*) or nidation* or fecund* or subfecund* or sub-fecund* or infecund* or infertil* or subfert* or sub-fert* or anovularit* or gonad* or reproduction* or Ovari* or ovary or ovulat* or folliculo* follicular or oogenes?s or oocyte* or parous or primipar* or multipar* or primigravid* or multigravid* or perinatal* or peripartum or peri-partum or prepartum or pre-partum or intrapartum or intra-partum or postpartum or post-partum or childbear* or ((vaginal or abdominal) NEAR/2 deliver*) or caesarean* or cesarean* or c-section* or embryo* or f?etus* or f?etal* or placenta* or prematur* or stillbirth* or abort* or congenital*):ti,ab,kw 266232

#10 [mh Reproduction] OR [mh ^"Pregnancy Outcome"] OR [mh ^"pregnancy rate"] 36485

#11 #7 OR #8 OR #9 OR #10 268116

#12 #3 AND #6 AND #11 291

#13 ([mh animal] OR [mh invertebrate] OR [mh ^"animal experiment"] OR [mh ^"animal model"] OR [mh plant] OR [mh fungus]) NOT [mh human] 3868

#14 #12 NOT #13 in Cochrane Reviews, Trials 291

**PRISMA 2020 flow diagram for new systematic reviews which included searches of databases and registers only**

**Identification of studies via databases and registers**

Records removed *before screening*:

Duplicate records removed with Deduklick (n = 1174)
Duplicate records removed with Covidence (n =5)

Duplicate records identified manually (n =1)

Records identified from*:

Medline (n = 2537)

Embase (n = 2037)
Cochrane (n = 291)

Total = (n = 4865)

**Identification**

Records excluded**

(n = 3607)

Records screened

(n = 3685)

Reports not retrieved

(n = 58)

Reports sought for retrieval

(n = 78)

**Screening**

Reports excluded:

Reason 1 (n = )

Reason 2 (n = )

Reason 3 (n = )

etc.

Reports assessed for eligibility

(n = 20)

Studies included in review

(n = )

Reports of included studies

(n = )

**Included**

*From:*  Page MJ, McKenzie JE, Bossuyt PM, Boutron I, Hoffmann TC, Mulrow CD, et al. The PRISMA 2020 statement: an updated guideline for reporting systematic reviews. BMJ 2021;372:n71. doi: 10.1136/bmj.n71
